# Chromosome-Specific DNA Repeats: Rapid Identification *in Silico* and Validation Using Fluorescence *in Situ* Hybridization

**DOI:** 10.3390/ijms14010057

**Published:** 2012-12-20

**Authors:** Joanne H. Hsu, Hui Zeng, Kalistyn H. Lemke, Aris A. Polyzos, Jingly F. Weier, Mei Wang, Anna R. Lawin-O’Brien, Heinz-Ulrich G. Weier, Benjamin O’Brien

**Affiliations:** 1 Department of Molecular and Cell Biology, University of California, Berkeley (UCB), Berkeley, CA 94720, USA; 2 Life Sciences Division, E.O. Lawrence Berkeley National Laboratory, 1 Cyclotron Road, Berkeley, CA 94720, USA; E-Mails: hzeng@lbl.gov (H.Z.); lemke.kalistyn@gmail.com (K.H.L.); aapolyzos@lbl.gov (A.A.P.); JLFung@lbl.gov (J.F.W.); ugweier@lbl.gov (H.-U.G.W.); 3 Department of Pathology, University of California, San Francisco (UCSF), CA 94143, USA; 4 Department of Diabetes, City of Hope, 1500 Duarte Road, Duarte, CA 91010-3012, USA; E-Mail: mwang@coh.org; 5 Department of Fetal Medicine, Whipps Cross University Hospital, Barts Health NHS Trust, London E11 1NR, UK; E-Mail: lawinobrien@doctors.org.uk; 6 William Harvey Research Institute, Queen Mary University London, Barts Health London, London EC1A 7BE, UK; E-Mail: benobrien@doctors.org.uk; 7 Department of Anesthesiology, German Heart Institute, Berlin 13353, Germany

**Keywords:** molecular cytogenetics, chromosomes, heterochromatin, DNA repeats, data mining, fluorescence *in situ* hybridization, bacterial artificial chromosomes, DNA probes

## Abstract

Chromosome enumeration in interphase and metaphase cells using fluorescence *in situ* hybridization (FISH) is an established procedure for the rapid and accurate cytogenetic analysis of cell nuclei and polar bodies, the unambiguous gender determination, as well as the definition of tumor-specific signatures. Present bottlenecks in the procedure are a limited number of commercial, non-isotopically labeled probes that can be combined in multiplex FISH assays and the relatively high price and effort to develop additional probes. We describe a streamlined approach for rapid probe definition, synthesis and validation, which is based on the analysis of publicly available DNA sequence information, also known as “database mining”. Examples of probe preparation for the human gonosomes and chromosome 16 as a selected autosome outline the probe selection strategy, define a timeline for expedited probe production and compare this novel selection strategy to more conventional probe cloning protocols.

## 1. Introduction

**Figure 1:**
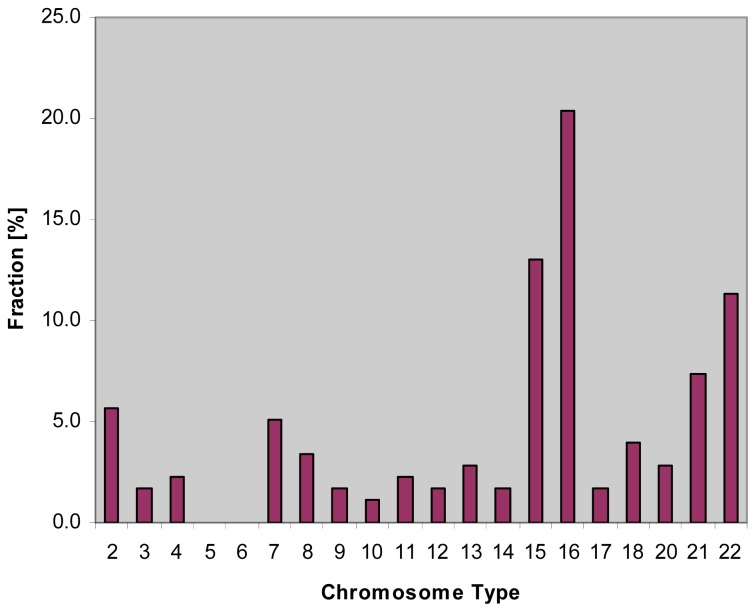
The percentage of chromosomal trisomies found in miscarriages (after Lathi *et al.*, Fertility and Sterility, 2008 [15] with permission).

Errors during mitotic cell division may lead to chromosome mis-segregation. Aneuploid daughter cells can have severe consequences, not only for the affected cell, but for an organism as a whole. Examples of this detrimental effect are the failure of aneuploid oocytes to fertilize [1], a reduced rate of the nidation of zygotes [2], a higher rate of spontaneously aborted embryos carrying a maternally derived supernumerary chromosome 16 (Figure 1) or the devastating consequences of trisomies on the development of human fetuses [3–11], only few of which survive to pregnancy term [12,13]. Furthermore, aneuploidy is associated with poor prognosis in solid tumors. Spontaneous chromosome mis-segregation events in aneuploid cells promote chromosomal instability that may contribute to the acquisition of multidrug resistance *in vitro* [14]. Therefore, different clinical settings, ranging from *in vitro* fertilization (IVF) and pre-implantation genetic diagnosis (PGD), perinatal analysis involving fetal and newborn tissues and the analysis of children with unexplained clinical symptoms to cancer research, have utilized a method called ‘Fluorescence *in situ* hybridization (FISH)’.

In a nutshell, FISH is based on the formation of stable hybrids between DNA targets inside cells and labeled DNA probes molecules provided by the investigator [16]. The DNA probes can either be marked by a fluorochrome, which can then be detected in the microscope, or by a non-fluorescent, non-isotopical hapten, most often biotin or digoxigenin, which is detected by a fluorescent moiety, such as fluorochrome-labeled avidin or antibodies against digoxigenin. Different probe types are available to suit particular applications: whole chromosome painting probes allow the delineation of inter-chromosomal translocations in metaphase spreads [17–19], while intra-chromosomal rearrangements are detected in metaphase or interphase cells with chromosome band-specific probes [20–23] or DNA probes targeting somewhat smaller, gene- or locus-specific regions [24–29].

While FISH found widespread application in research laboratories, its acceptance in clinical settings is mostly hampered by a limited selection of commercially available, U.S. Federal Drug Administration (FDA)-approved tests and the typically labor-intensive, costly effort to produce DNA probes that perform well in multiplexed assays [30]. Our laboratories have an established track record of production of novel DNA probes and innovative cell assays, many of which have found their way into contemporary cancer research or PGD analysis [18,21,23,24,26,31–38]. To facilitate the broad distribution of molecular cytogenetic assays and make DNA probes, as well as multiplex FISH tests, available to the less experienced laboratory, we have undertaken probe production pilot studies, which take advantage of the vast resources generated in the course of the Human Genome Project, such as physical maps and recombinant DNA libraries.

Our initial studies focused on the preparation of novel DNA probes for chromosome scoring or “enumeration” in interphase cell nuclei and metaphase spreads, since these seem to remain the most common applications in research and the clinic [30,39]. The vast majority of these chromosome enumerator probes (CEPs) target highly reiterated, tandemly-repeated DNA sequences in order to bind many copies of a rather small probe sequence to the target, which may be confined to a tightly localized area or volume. Different ways of isolating and purifying such DNA probes exist [31,37,38,40–45]. To the best of our knowledge, the procedures described in the present communication allow a laboratory with common equipment to prepare specific DNA probes in just a few days and, thus, represent the most efficient, rapid and cost-conscious approach to the generation of chromosome-specific DNA probes.

## 2. Results and Discussion

The development of probes that bind specifically to individual human chromosomes depends on the type of DNA repeat sequences identified on the target chromosome. Here, we will detail the approaches for preparation of probes for the human chromosomes Y, X and 16, since each of these chromosomes presented particular challenges.

### 2.1. Hybridization Targets on the Human Gonosomes

Two human gonosomes or “sex chromosomes” are carried in diploid cells as one copy each of the X- and Y-chromosome in male cells and two copies of the X-chromosome in female cells. In humans, where imprinting of gonosomal genes exists and dosage-compensation appears to exist for only a small subset of genes, the presence of an extra sex chromosome may lead to clinically recognizable phenotypes, including Turner and Klinefelter syndrome, hypogonadism, *etc.* [9,46,47]. Since gain or loss of a single gonosomes may not fully impair embryonic or fetal survival, prenatal screening procedures have kept a close watch on the sex chromosome make-up of cell specimens [48–50].

### 2.2. Hybridization Targets for Enumeration of Human Chromosome 16

**Figure 2:**
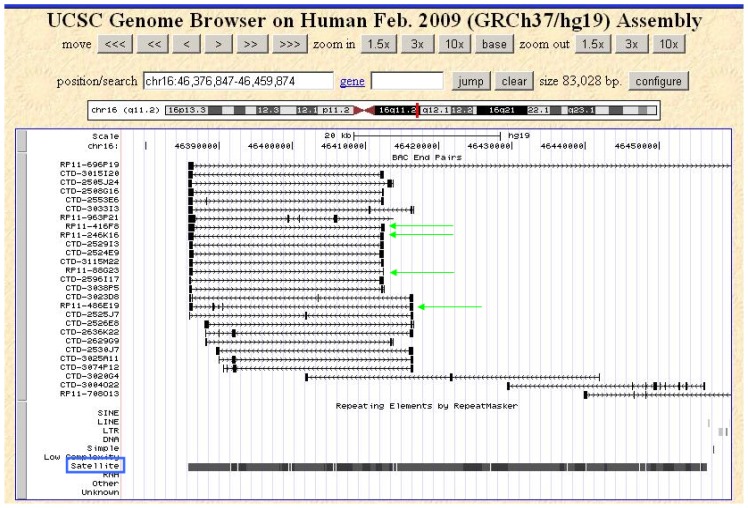
Selection of chromosome 16-specific, satellite DNA-containing bacterial artificial chromosome (BAC) clones. Bioinformatic analysis using RepeatMasker [51] indicated a region of chromosome 16 (vertical red bar in dark section in chromosome 16 ideogram, band 16q11.2 shown on top of the figure) that contains a dense assembly of tandemly repeated satellite DNA (blue box), but is free of other interspersed DNA repeats, which are not chromosome-specific. Four clones within this region were chosen for the present analysis (green arrows).

As mentioned earlier, the high frequency of spontaneous abortions with trisomy 16 [15] prompted our probe development. We chose the large block of DNA satellite II as a target for our probe development, since this heterochromatic block represents a very highly reiterated sequence (Figure 2).

The map positions of BAC discussed in this communication, as well as the BAC insert sizes and the Genbank accession numbers of insert end sequences, are listed in Table 1. The Y-specific BAC clone RP11-242E13 targets a highly repeated DNA sequence [52], while the X-specific clone RP11-294C12 targets a cluster of repeated alpha satellite DNA [38]. Thus, this probe combination is expected to bind to multiple sites along the long arm of the human Y chromosome and throughout the centromeric region of the X chromosome [31,38].

**Figure 3:**
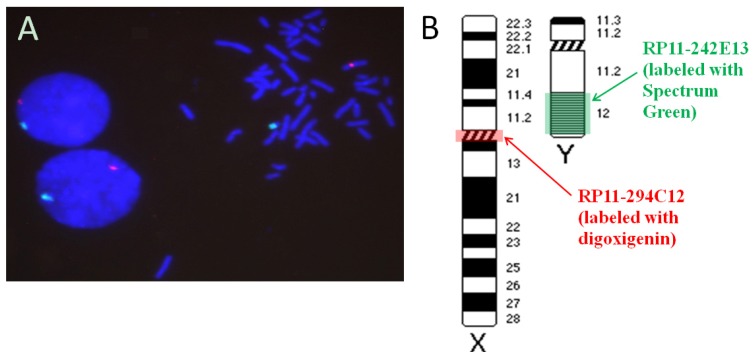
Results of *in situ* hybridization of chromosome X and Y BAC probes. (**A**) Dual color hybridization showing highly specific signals on the X (red) and Y (green) chromosomes in metaphase cells. The two diploid interphase cell nuclei from a normal male donor show the expected pair of single signals. (**B**) The approximate locations of the hybridization targets shown along ideograms of the human X and Y chromosomes.

The probes for the human X and Y chromosome labeled with digoxigenin and Spectrum Green, respectively, were used in a dual color FISH experiments. After incubation of the slide with rhodamine-conjugated antibodies against digoxigenin (Roche Molecular), the results showed unambiguous labeling of the target region on metaphase chromosomes and the expected number of red or green signals (Figure 3).

**Figure 4:**
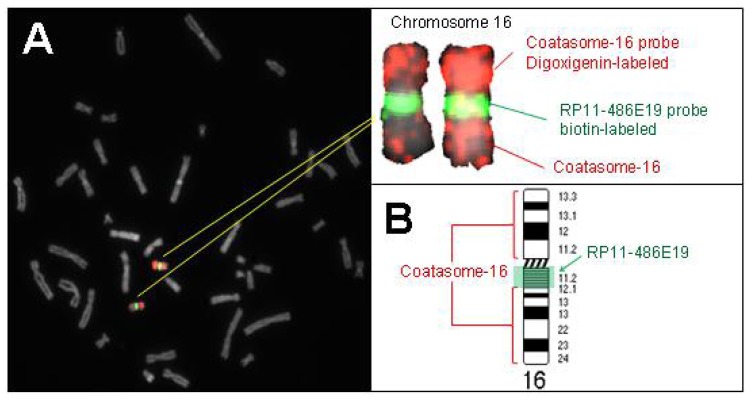
Results of *in situ* hybridization of a chromosome 16 BAC probe on metaphase spreads of ‘normal’ cells. (**A**) The dual color FISH results showing a normal diploid metaphase spread. The DAPI DNA counterstain is shown in gray; (**B**) Schematic diagram illustrating the relative positions of the chromosome 16 whole chromosome painting probe (Coatasome-16, Oncor) and the biotinylated DNA repeat probe prepared from BAC RP11-486E19 (detected with avidin-FITC, green).

**Figure 5:**
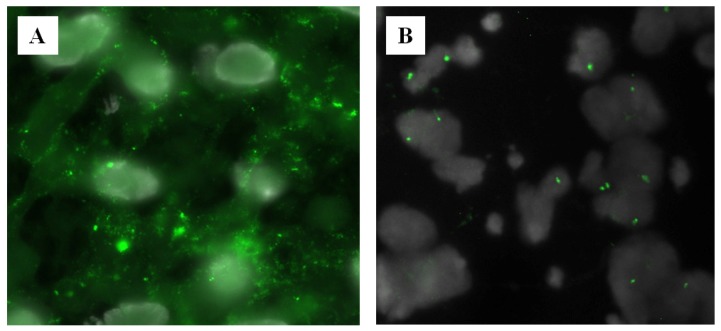
Hybridization of the chromosome 16-specific DNA probe to 8 μm thick tissue sections cut from human placenta tissue. (**A**) Without proteolytic pretreatment, tissue sections stored for about eight years at ambient temperature showed a large amount of unspecific probe binding; (**B**) A combined RNAse/pepsin pretreatment eliminated unspecific binding, thus greatly facilitating signal enumeration.

Results of the *in situ* hybridization of BAC-derived probes for chromosome 16 is shown in Figures 4 and 5 using metaphase spreads and tissue sections, respectively. Since the four selected probes showed virtually identical hybridization patterns, only one example is displayed here.

### 2.3. Clinical Perspective

Chromosomal aberrations are very frequently seen in human oocytes and embryos and are often responsible for poor pregnancy outcomes in natural, as well as assisted, conceptions [13].

Chromosome 16 trisomy carries specific significance, as it is considered the most common aneuploidy at conception [53], with an incidence of ~1.5% in all clinically recognized pregnancies [12]. Trisomy 16, arising from new non-disjunctional events, is the most common cause of sporadic first trimester miscarriage (Figure 1) and generally not compatible with life [54]. And, as the rate of trisomies increases with maternal age, the proportion of miscarriages with numeric chromosomal errors, such as trisomy 16, increases the older the mother is [55].

In assisted conception, *i.e.*, *in vitro* fertilization (IVF) and intracytoplasmatic sperm injection (ICSI), trisomy 16 or mosaic trisomy 16 in pre-implantation embryos are responsible for implantation failure, developmental arrest and miscarriage. Here, pre-implantation genetic diagnosis (PGD) represents a significant scientific advance for couples at risk of having children with heritable and debilitating genetic diseases. For couples who carry a balanced chromosomal translocation [25,56,57], PGD significantly decreases the risk of spontaneous miscarriage and significantly increases live-birth rates [58,59]. The technique described in this communication can be adapted to detect balanced translocations, when required by parents faced with such a problem. PGD algorithms often also include important new technologies, such as arrayCGH, for the detection of aneuploidy, balanced translocations and other chromosome anomalies [59].

Pre-implantation embryos not only present meiotically derived aneuploidy, but also post-zygotic chromosome segregation errors in the cleavage stage [56]. Mitotic chromosome mal-segregation may lead to exaggerated mosaicism, with a minority of embryos displaying a mixture of normal and aneuploid cells and most mosaic embryos presenting two or more abnormal cell lineages. Some embryos show failure of cellular mechanisms that control accurate chromosome segregation, becoming karyotypically unstable or “chaotic mosaics” [56,60]. Aneuploidy screening for embryo selection in assisted conception is developing fast, as it translates into improved clinical outcomes for infertile patients [61–63].

## 3. Experimental Section

### 3.1. Choosing DNA Probes That Bind to Specific Human Chromosomes

#### 3.1.1. Selection of Probes for Satellite-Rich Regions of Human Chromosomes 16 and X

The UCSC Genome Browser GRCh37/hg19, built February 2009, was used to identify bacterial artificial chromosome (BAC) clones with high satellite DNA content in the non-centromeric and non-telomeric regions of the short and long arms of human chromosomes 16, X and Y [38]. The graphic user interface was set to display BAC end pairs and repeat DNA elements. A region with high satellite content was identified from which BAC probes were then chosen (*i.e.*, chromosome 16: 46,385,500 bp-46,457,245 bp, chromosome X: 58,232,531 bp-61,922,800 bp).

#### 3.1.2. Determining the Target Sequence on the Y Chromosome

The nucleic acid sequence used for data mining was defined in our previous studies on *in vitro* DNA amplification of Y chromosome-specific DNA repeat sequences. Specifically, we designed pairs of oligonucleotide primers to amplify stretches of 124 bp from the 3.6 kb pentanucleotide DNA repeat described by Nakahori *et al.* (DYZ1, Genbank accession number X06228) [31,52]. Primer annealing sites were chosen to have minimal homology with the human satellite III DNA repeat consensus sequence “TTCCA” [64,65]. Blood samples from six normal human volunteers were used to validate the Y chromosome-specific PCR assay [66,67].

Serial cell dilution experiments and artificial mixing of flow-sorted Y chromosome carrying cells in predetermined aliquots of white blood cells from female donors determined that the primer combination WYR 4 (5′-GAACCGTACGATTCCATTCCTTTTGAA-3′)–WYR 6 (5′-TTCCATTCCATTCCATTCCTTTCCTTT-3′), amplifying a 248 bp DNA fragment corresponding to position 2965–3212 in Genbank accession number X06228, was sufficiently specific to detect a single male cell in the background of 1 million female cells [68]. Samples comprised entirely of female cells did not yield this product [66,67].

#### 3.1.3. Identifying a DNA Probe for the Target Sequence of Chromosome Y

We screened the human genome nucleotide DNA database at the NCBI for homologous sequences using one of the most widely used bioinformatics programs, Basic Local Alignment Search Tool (BLAST) [69]. The BLAST approach to rapid sequence comparison directly approximates alignments that optimize a measure of local similarity, the maximal segment pair score. The basic algorithm is simple, robust and versatile; it can be implemented in a number of ways and applied in a variety of contexts, including straightforward DNA and protein sequence database searches, motif searches, gene identification searches and in the analysis of multiple regions of similarity in long DNA sequences [69,70].

Execution of the BLAST querying the human genome database with the 27-nucleotide (nt) sequence “ATTCCGTACGATTCCATTCCTTTTGAA” from position 3089–3115 of the human Y-specific 3564 bp repeat (Genbank accession number X06228), performed at the NCBI web site [71], retrieved multiple hits. Parameters were set to identify clones with a range of levels of nucleic acid homology (setting: “Optimize for somewhat similar sequences”, (BLASTn)).

### 3.2. Fluorescence *in situ* Hybridization

Individual BAC clones were grown overnight in up to 10 mL of Luria broth (LB) medium containing 12.5 μg/mL chloramphenicol (Sigma, St. Louis, MO, USA), and the DNA was isolated using a ZR BAC DNA Miniprep Kit (Zymo Research, Irvine, CA, USA). For the preparation of DNA pools, clones were grown individually and pooled prior to DNA isolation. The isolation of high molecular weight BAC DNAs was confirmed on 1% agarose gels and quantitated by spectrophotometry (Nanodrop 2000, Thermo Scientific, Wilmington, DE, USA). Probe DNAs were labeled with biotin-14-dCTP (part of the BioPrime kit, Invitrogen, La Jolla, CA, USA), digoxigenin-11-dUTP (Roche Diagnostics, Indianapolis, IN, USA) or Spectrum Green-dUTP (Abbott, Downers Grove, IL, USA) by random priming using a commercial kit (BioPrime Kit, Invitrogen) [23]. When incorporating fluorochrome-labeled deoxynucleoside triphosphates, such as Spectrum Green-dUTP, the dTTP to dUTP ratio in the reaction was adjusted to 2:1 [26,72–74].

Between 0.5 μL and 1 μL of each probe, along with of 0.25 μL human COT1™ DNA (1 mg/mL, Invitrogen) and 0.25 μL salmon sperm DNA (20 mg/mL, 3′-5′, Boulder, CO), were added to 3.9 μL of hybridization master mix (50% formamide (FA), 20% dextran sulfate, in 2 × SSC and 50mM phosphate buffer pH 7.0) in a total volume of 6.9 μL. Hybridization and detection of bound probes followed our published procedures [37,44,75,76]. Biotinylated and digoxigenin-labeled probes were detected with avidin-fluorescein isothiocyanate (FITC) (Vector, Burlingame, CA, USA; green fluorescence) and rhodamine-conjugated antibodies against digoxigenin (Roche Diagnostics; red fluorescence).

### 3.3. Pretreatment of Tissue Sections with RNase and Pepsin

A pretreatment of the slides with RNase and pepsin followed by post-fixation with formalin buffer was required to reduce the background. Slides were soaked in 2 × SSC for 5 min at 21 °C on a shaking platform (20 × SSC is 3 M sodium chloride and 300mM tri-sodium citrate, pH 7.0). Slides were then placed into a Coplin jar with RNase solution (RNase solution: 50 μg/mL in 2 × SSC) and incubated for 15 min at 37 °C, then washed with 2 × SSC for 3 min on a shaker. Tissue sections were then treated with pepsin-buffer at 37 °C for 20 min (without agitation) (pepsin buffer: freshly prepared 50 μg/mL pepsin in 0.01 M HCl, pre-warmed to 37 °C). Sections were then washed twice for 5 min with 50 mM MgCl_2_ (in 1 × PBS) at 21 °C before they were dehydrated in an ethanol series (70%, 85%, 100%; 3 min each) and air dried.

### 3.4. Image Acquisition and Analysis

Fluorescence microscopy was performed on a Zeiss Axioskop or Axiovert.A1 microscope (Carl Zeiss GmbH, Oberkochen, Germany) equipped with a quadruple filter set for single wavelength excitation and observation of either DAPI, FITC, Texas Red/rhodamine/Cy3.5 or Cy5/Cy5.5 fluorescence (84000v2 Quad, ChromaTechnology, Brattleboro, VT, USA). Images were collected using an Axiocam HR camera (Carl Zeiss GmbH), and image processing was done using the Axiovision software (Carl Zeiss GmbH) or Photoshop software (Adobe, Inc., Mountain View, CA, USA).

## 4. Conclusions

For the fetal medicine specialist and neonatologists, greater clinical significance lies with the few embryos carrying a rare trisomy 16 mosaic aberration, which do not die in utero. “Confined placental mosaicism” (CPM) is used to describe trisomy 16 mosaicism, as the trisomic cells are predominantly confined to the placenta [46,77,78]. CPM mosaicism for trisomy 16 diagnosed at amniocentesis or chorion villous biopsy can bear a great dilemma for genetic counseling, as the pregnancy outcome is overall poor, but difficult to predict [53,54]. Among the fetuses reaching viability, up to 45% have at least one malformation, predominantly congenital heart defects, but also pulmonary, genitourinary, gastrointestinal or craniofacial anomalies. There is a significant increased risk of severe intrauterine growth restriction and preterm delivery, with consecutively high perinatal morbidity and mortality [79].

Irrespective of the clinical context, this state-of-the-art technique for identification, preparation and hybridization of FISH probes has the potential to take the feasibility and applicability of clinical investigations utilizing chromosome-specific DNA repeats to a whole new level [7,30]. At last, routine testing, as well as larger-scale studies, can be affordable, reliably delivered and readily adapted by smaller, clinical, non-academic laboratories.

In addition, since cells deriving from a failed trisomy 16 pregnancy are unique and readily identifiable, it would be feasible and of interest to use tailor-made probes, as described in this communication, to search for persistent fetal cells in maternal peripheral blood. The fate of these cells or, indeed, their significance is not yet understood.

It is important to note that the use of BAC clones for probe preparation is significantly faster and more efficient than the preparation of probes from yeast artificial chromosomes [80]. In our experience, the clones can be grown much faster, and BAC-derived probes need little or no optimization, so that the cycle from probe selection to hybridization results is reduced to less than half the time [45].

## Figures and Tables

**Table 1:** Selection of BAC clones specific for satellite-rich regions of chromosomes X, Y and 16.

BAC	Chr.	Band	Start (bp) *	End (bp) *	Insert (bp)	BAC End Sequence Accession Number
RP11-242E13	Y	q12	multiple	multiple	98295	AC068123
RP11-348G24	X	p11.1	58,356,061	58,564,667	208607	AQ528470, AQ528473
RP11-88G23	16	q11.2	46,385,822	46,412,445	26624	AQ285754, AQ285753
RP11-246K16	16	q11.2	46,385,808	46,412,485	26678	AQ478385, AQ478388
RP11-416F8	16	q11.2	46,385,808	46,412,485	26678	AQ551230, AQ661227
RP11-486E19	16	q11.2	46,385,921	46,412,470	26550	AQ629869, AQ629871

Note:

* Data obtained from the National Center for Biotechnology Information (NCBI), National Institute of Health, mapviewer page, Build 37.2.
